# Classification of Clouds in Satellite Imagery Using Adaptive Fuzzy Sparse Representation

**DOI:** 10.3390/s16122153

**Published:** 2016-12-16

**Authors:** Wei Jin, Fei Gong, Xingbin Zeng, Randi Fu

**Affiliations:** 1 Faculty of Electrical Engineering and Computer Science, Ningbo University, Ningbo 315211, China; jinwei@nbu.edu.cn (W.J.); furandi@nbu.edu.cn (R.F.); 2 Intelligent Household Appliances Engineering Center, Zhejiang Business Technology Institute, Ningbo 315012, China; zengxingbin_zxb@126.com

**Keywords:** satellite imagery, cloud classification, adaptive fuzzy membership function, adaptive fuzzy sparse representation

## Abstract

Automatic cloud detection and classification using satellite cloud imagery have various meteorological applications such as weather forecasting and climate monitoring. Cloud pattern analysis is one of the research hotspots recently. Since satellites sense the clouds remotely from space, and different cloud types often overlap and convert into each other, there must be some fuzziness and uncertainty in satellite cloud imagery. Satellite observation is susceptible to noises, while traditional cloud classification methods are sensitive to noises and outliers; it is hard for traditional cloud classification methods to achieve reliable results. To deal with these problems, a satellite cloud classification method using adaptive fuzzy sparse representation-based classification (AFSRC) is proposed. Firstly, by defining adaptive parameters related to attenuation rate and critical membership, an improved fuzzy membership is introduced to accommodate the fuzziness and uncertainty of satellite cloud imagery; secondly, by effective combination of the improved fuzzy membership function and sparse representation-based classification (SRC), atoms in training dictionary are optimized; finally, an adaptive fuzzy sparse representation classifier for cloud classification is proposed. Experiment results on FY-2G satellite cloud image show that, the proposed method not only improves the accuracy of cloud classification, but also has strong stability and adaptability with high computational efficiency.

## 1. Introduction

Meteorological satellites have advantages such as a wide range of spatial observations, high temporal resolution, all-weather observation, etc. Satellite cloud imagery has become one of the important means for weather forecasting and climate analysis [1,2,3], especially for forecasting and monitoring some natural disasters, such as typhoons, floods, snowstorms, forest fires, etc. Cloud classification is one of the fundamental works of satellite cloud image processing. The current commonly used methods for cloud classification are threshold based methods (including simple threshold method and histogram based method mainly), statistical based methods, and artificial intelligence based methods [4]. Simple threshold methods [5,6] analyze the spectral characteristics of different channels to inverse the brightness temperature for each pixel, then take the gray value and brightness temperature of each pixel with the difference of brightness temperature between channels to determine a series of thresholds as comprehensive criterion, but it is very difficult to determine the series of thresholds. Histogram based method [7] improves the simple threshold method by taking advantage of statistical properties of the partial or global histograms of satellite cloud images, but for complex weather systems, it is still hard to obtain adaptive thresholds. As for the statistical-based methods—such as C-means, fuzzy C-means clustering [8], etc.—based on the cluster analysis of sample set, these methods classify the pixels into different types of clouds by computing the distance between samples and cluster centers. The performance of cloud classification could be improved, but in case that different clouds superimposed each other, their clustering results is poor, thus they cannot achieve reasonable classification performance. In recent years, artificial intelligence based methods, such as artificial neural networks [9] and support vector machine (SVM) [10,11,12,13,14,15], have been applied to cloud classification. These methods improved the accuracy of cloud classification by using machine learning to extract the inherent features of training samples. In particular, support vector machine, which is based on the principle of structural risk minimization and Vapnik-Chervonenkis (VC) dimension, can find the best compromise between learning ability and model complexity, thus it can obtain good generalization ability for cloud identification. However, actual weather systems are really intricate; the distributions of clouds are very perplexing. For example, in transition regions of different cloud types, and in regions where the clouds are just in developing or extinction phases, there are not only clouds with distinctive features, but also some clouds with fuzzy properties. Even worse, satellite cloud imagery may easily be deteriorated by noises, atmospheric turbulence, fluctuation of undulating surface or drift of satellite orbit, etc. So it is inevitable that cloud images possess the characteristics of fuzzy, non-uniform, complex, and changeable cloud types. Thus, introducing fuzzy theory into satellite image processing may improve the accuracy of cloud classification. Based on traditional SVM, using linear fuzzy membership to optimize the sample set, Lin et al. constructed a fuzzy support vector machine (FSVM), experiments showed that FSVM can reduce the sensitivity to noises and outliers of traditional SVM [12]. Fu et al. applied a modified FSVM to the detection of cumulus cloud, which achieves good performance [13], but it cannot detect other cloud types effectively, and its generalization is relatively poor. To handle the deficiency of traditional FSVM, Zhang et al. defined affinity among samples and proposed an affinity based FSVM [14]. According to the affinity among samples, the modified FSVM assigns each sample a proper fuzzy membership to suppress the impact caused by noises and outliers during the training of SVM, and presents an effective scheme to identify valid or invalid samples. However, the definition of the fuzzy membership function based on affinity cannot accurately depict the changing trends of different samples; moreover, the function parameters are not very adaptable or flexible. 

Due to the successful application of sparse representation for pattern classification problem [16,17,18], our research team proposed a cloud classification method using over-complete dictionary via sparse representation [19], which improved the classification accuracy in most weather systems, but this method did not take the fuzziness of cloud samples into consideration. By introducing the fuzziness into sparse representation, a cloud classification method using adaptive fuzzy sparse representation-based classification (AFSRC) is proposed, this method defines some adaptive parameters which relate to the attenuation rate and critical value of fuzzy membership, then constructs an adaptive membership function to alleviate the problem that the traditional fuzzy membership function is ineffective in depicting the distribution characteristics of training samples. Finally, the proposed fuzzy membership function is combined with sparse representation to design an adaptive classifier. The motivation of this method lies in that a single pixel of cloud image is usually the comprehensive reflection of different clouds and surface features; it implies that a single pixel is a linear combination of several components of cloud systems in a picture, which exactly coincide with the idea of sparse representation [16], so sparse representation has some promising applicability for cloud classification. Moreover, by introducing an adaptive fuzzy membership function into sparse representation to optimize the sample set, the adaptive dictionary with stronger discriminative capability can be obtained, which is expected to improve the accuracy of cloud classification.

The rest of the paper is organized as follows: Section 2 briefly introduces the cloud feature extracting and cloud classification system for satellite imagery; Section 3 introduces the traditional fuzzy membership theory for cloud classification; Section 4 presents the proposed adaptive fuzzy membership function, and combines it with sparse representation classifier, providing the proposed scheme for cloud classification; Section 5 gives the experiment results; the conclusion is presented in Section 6.

## 2. Satellite Data and Cloud Classification System

### 2.1. Satellite Data Feature Extraction 

This paper takes FY-2G satellite cloud image as the data source. The scanning radiometer carried by FY-2G satellite has five imaging channels, which includes two infrared long wave channels (IR1, 10.3~11.3 μm; IR2, 11.5~12.5 μm), one water vapor channel (IR3, 6.3~7.6 μm), one infrared medium wave channel (IR4, 3.5~4.0 μm), and one visible spectrum channel (VIS, 0.55~0.75 μm). Since different imaging channels reflect atmospheric physics parameters such as top brightness temperature, albedo, and water vapor content in different aspects, the comprehensive data have brought great convenience for various meteorological applications. By the information of these channels, the inversion accuracy of clouds and underlying surface can be greatly improved.

Due to the close relationship between cloud data of infrared channels and infrared radiation of clouds and the underlying surface in infrared cloud images (IR1, IR2, IR4) of FY-2G, most dark areas often indicate the highest temperature areas such as clear land, clear water such as lakes, and oceans in terms of temperature. Contrary to the dark areas, the bright white areas in infrared imagery often indicate different types of cloud, and clouds usually have a relatively low temperature. What is more, infrared channel data can not only indicate the height of clouds, but can also help in distinguishing different cloud types, land, and ocean from each other. The water vapor channel (IR3) is a special infrared band; vapor channel data indicate the absorption of infrared radiation by water vapor. The more water vapor the atmosphere contains, the more infrared radiation will be absorbed, and this makes the corresponding area in water vapor imagery whiter; thus, water vapor imagery can help to estimate the content of water vapor and also help to classify different cloud types. As for the visible spectrum channel, data of VIS indicates the albedo of solar radiation. So, generally, darker areas in VIS cloud imagery indicate clear sky and brighter areas indicate different cloud types for their higher albedo. Furthermore, the albedo is positively related to the thickness of cloud, so the VIS cloud image often shows sharper contrast in brightness, thus it also provides effective information for classification of different cloud types.

**Figure 1:**
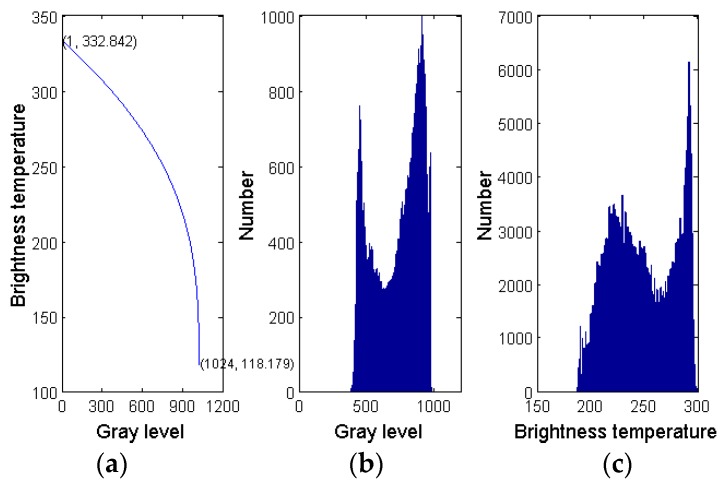
Analysis diagram of gray and brightness temperature. (**a**) Nonlinear curve for gray value and brightness temperature of IR1 image; (**b**) Gray histogram for IR1 image; (**c**) Top brightness temperature histogram of IR1 image.

In order to classify different cloud types effectively, the gray values of each pixel in the cloud images from five channels are selected as basic features. Considering that although gray value of infrared and VIS cloud images indicate the top brightness temperature or albedo of clouds to some extent, extracting brightness temperature and albedo may help in classifying different cloud types. However, the gray value of pixels is nonlinear with the top brightness temperature and albedo. According to the imaging properties of a scanning radiometer for FY-2G, cloud top brightness temperature of infrared image and albedo of VIS image are also extracted as extra features of samples. Figure 1a shows the relationship of brightness temperature versus gray value of IR1 cloud image provided by FY-2G, Figure 1b shows the gray value histogram of IR1 cloud image, and Figure 1c shows the brightness temperature histogram of an IR1 cloud image. It can be seen that the brightness temperature and gray value have different distribution characteristics, thus brightness temperature is taken as a component of the feature vector, which strengthens the discriminative ability of the feature vector, and makes the sample feature more representative. For other infrared and VIS channels, their brightness temperature and albedo are extracted by the same method. In addition, the brightness temperature difference between different infrared channels indicate the radiation characteristics of different clouds [9], for example, the brightness temperature difference between IR1 and IR2 can be used to identify cirrus and cumulonimbus, and there is a strong correlation between the top height of convective cloud and the brightness temperature difference between IR1 and IR3, etc. Thus, in this paper, brightness temperature difference IR1-IR2, IR1-IR3, IR1-IR4, and IR2-IR3 are extracted as additional features for cloud classification. Table 1 lists the components of feature vectors for cloud classification; Table 2 briefly describes the main identification characteristics of different components.

The above mentioned 14 features were used to construct the original samples, denoted as $x=(x_{1},x_{2},\ldots ,x_{14})$, and then it was normalized by $\ell_{2}$-norm. The $\ell_{2}$-norm of **x** is defined as norm $(x)=\sqrt{x_{1}^{2}+x_{2}^{2}+\ldots +x_{14}^{2}}$, and the normalized sample vector is $x^{\prime }=(x_{1}^{\prime },x_{2}^{\prime },\ldots ,x_{14}^{\prime })$, where $x_{i}^{\prime }=\frac{x_{i}}{\text{norm}(x)},i=1,2,\ldots ,14$.

### 2.2. Cloud Classification System

The satellite cloud images, recorded by infrared and visible channel, mainly reflect the top brightness temperature and albedo information of clouds; it is difficult to grasp the generation procedure of clouds, and to analyze the cloud particles. Generally speaking, each pixel in cloud images is a comprehensive reflection of different clouds and underlying surface, thus it is hard to achieve an accurate classification of satellite cloud imagery. Now, following the international common practice of cloud classification, according to their height and vertical development, clouds are mainly divided into four families as high cloud, medium cloud, low cloud, and heap cloud [4]. The four families are subdivided into several categories: high cloud is subdivided into cirrus, cirrostratus, and cirrocumulus; medium cloud is subdivided into altostratus and altocumulus; low cloud is subdivided into cumulus, stratus, stratocumulus, and nimbostratus; and heap cloud mainly refers to cumulonimbus.

Following the specific requirements of satellite cloud image classification for meteorological services, in this paper, each satellite cloud image pixel is classified as one of the following six types: clear water, clear land, heap cloud, low cloud, medium cloud, and high cloud. Since cumulus, stratus, nimbostratus, and stratocumulus are mainly made up of water drops and they frequently bring continuous rain, they are all classified as low clouds. Similarly, altostratus and altocumulus are just classified as medium clouds; cirrus, cirrostratus, and cirrocumulus, generally made up of ice crystals, with a cloud base height of usually more than 5000 m, and which generally do not bring precipitation, are classified as high clouds; for heap cloud, cumulonimbus are the focus, because its cloud top may extend to the scope of a medium-level or even high-level cloud, which reflects a strong updraft, and they usually incur severe convection weather as thunderstorms or heavy rainfall, so cumulonimbus attracts attention in meteorological monitoring.

## 3. Fuzzy Membership for Cloud Classification

In actual weather systems, apart from the clouds with distinguishing characteristics, there are also some clouds with fuzzy characteristics; thus, it is difficult to classify cloud into a specific cloud type arbitrarily. Establishing a fuzzy membership to describe the relation between samples and specific cloud types is a helpful scheme. In fact, in the application of machine learning, fuzzy membership plays an important role in depicting the relations among training samples and specific classes. If training sample $x_{i}$ is assigned a fuzzy membership $p_{i}\in [0,1]$, which denotes its specific belonging class, a soft classification model can be established and the classification performance of machine learning algorithms can be improved. Since traditional SVM is sensitive to noises or outliers, Lin et al., by assigning smaller membership to noises or outliers to eliminate their impact on hyperplane of classification, proposed the fuzzy support vector machine (FSVM) and improved the performance of SVM classifier effectively [12]. So, constructing a reasonable fuzzy membership function is the key to building a soft classification model. 

**Figure 2:**
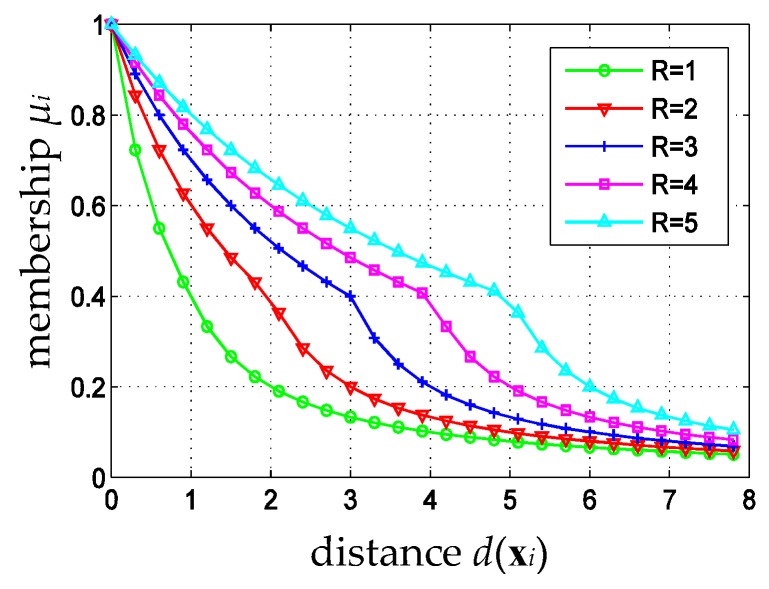
Membership function curve of Equation (1) for different R.

The traditional linear and sigmoid membership functions cannot depict the distribution characteristic of invalid samples such as noises and outliers effectively, and they cannot reflect the uncertainty of samples. To solve the problem, Ref. [14,15] proposed a membership function based on affinity. A minimum hypersphere is constructed, which contains most of the valid samples, and the affinity among samples, defined by support vector data description (SVDD) [20], is represented by the radius of the minimum hypersphere. The membership function is then constructed as follows [14]:

(1) $$\mu_{i}=\{\begin{array}{ll} 0.6\times (\frac{1-\frac{d(x_{i})}{R}}{1+\frac{d(x_{i})}{R}})+0.4, & d(x_{i})\leq R\\ 0.4\times (\frac{1}{1+(d(x_{i})-R)}), & d(x_{i})>R \end{array}$$

here, $d(x_{i})(i=1,2,\ldots ,l)$ is the distance between training samples $x_{i}$ and its class center, *R* is the radius of the minimum hypersphere. If $d(x_{i})\leq R$, which means that $x_{i}$ lies inside the hypersphere, or $x_{i}$ is more likely to be a valid sample, the corresponding membership of the sample is calculated using the upper formula of Equation (1). Otherwise, if $d(x_{i})\geq R$, which means that $x_{i}$ lies outside the hypersphere, the corresponding membership of the sample is calculated using the lower formula of Equation (1). According to the definition of membership function as Equation (1), the membership is larger than 0.4 for samples inside the hypersphere, and is less than 0.4 for samples outside the hypersphere; here, 0.4 acts as a critical membership. By calculating membership with different formula for samples inside or outside the hypersphere, the membership function could distinguish valid and invalid samples better and decrease the membership for noises and outliers, so as to eliminate their adverse impact on the classification hyperplane. Figure 2 shows a set of membership function curves of Equation (1) for different *R*. 

Figure 2 shows that the critical membership depicts the boundary between the inner and outer samples of the minimum hypersphere, and the fuzzy memberships vary differently for different radius *R*. As *R* increases, the membership attenuation rate for samples outside the hypersphere (where $\mu_{i}<0.4$ in Figure 2) accelerates a bit, while that slows down for samples inside the hypersphere (where $\mu_{i}>0.4$ in Figure 2). If *R* is too small (R<3), the membership attenuation rate for samples outside the hypersphere is smaller than that for samples inside the hypersphere. In this paper, a 14-dimensional feature vector is extracted and then normalized. After normalization, for real data, the radius *R* of the minimum hypersphere for different cloud type sample sets are all less than 3. In practice, samples inside or outside the hypersphere are treated as valid or invalid samples, respectively. To show the different importance of valid or invalid samples in designing a classifier, the membership should attenuate slowly for valid samples, and should attenuate far more quickly for invalid samples. Hence, the membership function defined by Equation (1) is not consistent with the distribution characteristics of satellite cloud images, which will degrade the performance of cloud type identification. In addition, the critical membership in Equation (1) is fixed to 0.4, which is not good enough. In fact, different sample sets might have different distribution characteristics, and their corresponding critical membership should be different, too. In the following section, membership function with adaptive parameters will be defined, and an adaptive fuzzy sparse representation classifier for cloud type identification will be constructed.

## 4. Adaptive Fuzzy Sparse Representation Classifier for Cloud Type Identification

In actual weather systems, cloud distribution is very complex; clouds may overlap each other and are changing all the time; noises and data errors in acquisition and transmission make it more difficult to conduct satellite cloud image processing. In order to achieve better classification performance, it is necessary to utilize the distribution characteristics of training samples. After analyzing the distribution characteristics of training samples for cloud classification, by eliminating the influence of outliers and noises, an adaptive fuzzy membership function is designed to improve the capability of affinity-based fuzzy membership function. Combining the improved fuzzy membership function with the sparse representation classifier, an adaptive fuzzy sparse representation classifier is constructed for cloud type identification.

### 4.1. Adaptive Fuzzy Membership Function

As mentioned above, since there are many noises and outliers in cloud training sample sets, it is difficult for the affinity-based fuzzy membership function to handle them. To make the membership function better for the classification of clouds in satellite cloud image, three new parameters are introduced. Parameters $\rho_{I}(0<\rho_{I}<1)$ and $\rho_{o}(\rho_{o}>1)$ are used to control the membership attenuation rate for samples inside or outside the hypersphere, respectively, and $\hat{\mu }(0<\hat{\mu }\leq 1)$ is used to control the critical membership. The modified membership function is defined as

(2) $$\mu_{i}=\{\begin{array}{ll} (1-\hat{\mu })\times {\left(1-\frac{d(x_{i})}{R}\right)}^{\rho_{I}}+\hat{\mu }, & d(x_{i})\leq R\\ \hat{\mu }\times {\left(\frac{1}{1+(d(x_{i})-R)}\right)}^{\rho_{o}}, & d(x_{i})>R \end{array}$$

**Figure 3:**
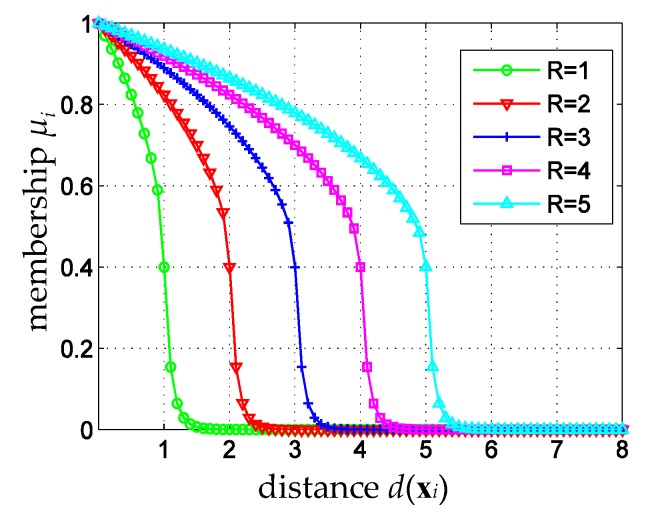
The modified membership function with $\rho_{I}=0.5$, $\rho_{o}=10$, and $\hat{\mu }=0.4$.

As a demo, the modified membership function curves with $\rho_{I}=0.5$, $\rho_{o}=10$, and $\hat{\mu }=0.4$, are shown in Figure 3. It is clear that the modified membership function not only inherits the merit of affinity-based fuzzy membership function that the minimum hypersphere gives a clear boundary between valid and invalid samples, but also shows the advantage of sigmoid membership function that the membership attenuation rate for valid samples is slower than that for invalid samples.

According to Equation (2), parameters $\rho_{I}(0<\rho_{I}<1)$, $\rho_{o}(\rho_{o}>1)$, and $\hat{\mu }$ are involved in calculating the modified membership for each sample. Though these three parameters can be obtained by experiment, they can be determined adaptively according to the distribution of actual samples, and in turn the fuzzy membership function constructed will be adaptive, too. The three parameters are determined as follows:

1. Membership attenuation rate $\rho_{I}$ for samples inside the hypersphere

**Figure 4:**
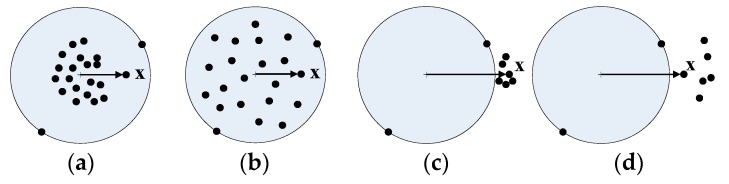
Four different types of samples distribution inside/outside the hypersphere. (**a**) Type 1, most samples are more condensed to the center of the hypersphere; (**b**) Type 2, samples distribute more randomly over the hypersphere; (**c**) Type 3, some samples outside the hypersphere are more condensed; (**d**) Type 4, samples outside the hypersphere distribute more randomly.

Figure 4a,b show two different types of samples distribution inside the hypersphere. The distances between the sample **x** and its center in Figure 4a,b are the same. If parameter $\rho_{I}$ corresponding to sample **x** in Figure 4a,b were set to be the same, their membership would be the same too. It is clear that, those samples in Figure 4a are more condensed to the center of the hypersphere, and those samples in Figure 4b distribute more randomly over the hypersphere, so the membership of sample **x** in Figure 4b should be larger than that in Figure 4a. Therefore, $\rho_{I}$ with the same value in Types 1 and 2 is not reasonable, $\rho_{I}$ should be related to sample distributions inside the hypersphere. The closer to the hypersphere surface the samples distribute, the slower the membership attenuates, and the smaller $\rho_{I}$ should be.

Accordingly, the average radius $d_{I}(d_{I}\leq R)$ between samples and their class centre is defined to describe the overall distribution of samples inside the hypersphere.

(3) $$d_{I}=\frac{\sum_{i=1}^{n_{I}}d(x_{i})}{n_{I}},d(x_{i})\leq R$$

where $n_{I}$ is the number of samples inside the hypersphere and $\rho_{I}$ can be defined as

(4) $$\rho_{I}=1-\frac{d_{I}}{R}$$

**Figure 5:**
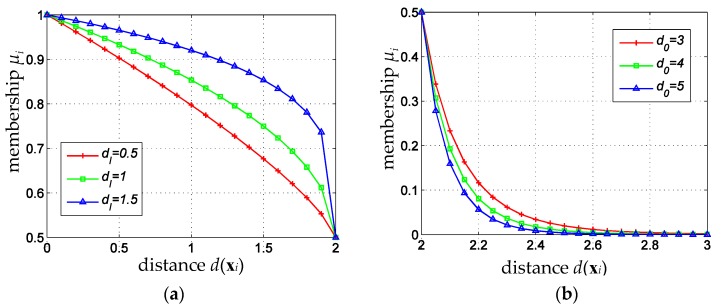
Membership curves corresponding to different $d_{I}$ and $d_{o}$. (**a**) Membership curves to different $d_{I}$; (**b**) Membership curves to different $d_{o}$.

Figure 5a shows memberships for samples inside the hypersphere with $\rho_{I}$ as Equation (4), *R* = 2 and $\hat{\mu }=0.5$. It is clear that if more samples inside the hypersphere are close to the surface, the membership attenuates more slowly.

2. Membership attenuation rate $\rho_{o}$ for samples outside the hypersphere

In Figure 4c,d, the distances between sample **x** outside the hypersphere and its center are the same. If the distribution characteristic of samples outside the hypersphere was ignored, the same value of $\rho_{o}$ was used, then the membership for sample **x** in Figure 4c,d would be the same. To differ the membership for sample **x** in Figure 4c from that in Figure 4d, the average radius $d_{o}(d_{o}>R)$ between samples and their class center is defined as Equation (5), which describes the overall distribution of samples outside the hypersphere.

(5) $$d_{o}=\frac{\sum_{i=1}^{n_{o}}d(x_{i})}{n_{o}},d(x_{i})>R$$

where $n_{o}$ is the number of samples outside the hypersphere and $\rho_{o}$ is defined as

(6) $$\rho_{o}=K\times \frac{d_{o}}{R}$$

After some tests, we found that *K* = 5 gave the best results. Figure 5b shows memberships for samples outside the hypersphere with $\rho_{o}$ as Equation (6), *R* = 2 and $\hat{\mu }=0.5$. It is clear that if most samples outside the hypersphere are close to the surface, the membership attenuates more slowly.

3. Critical membership $\hat{\mu }$

The critical membership $\hat{\mu }$ means the minimum membership for samples inside the hypersphere and the maximum membership for samples outside the hypersphere. Figure 4 shows that, the closer to the surface the samples outside the hypersphere lie, the more possible it is that these samples belong to the class, and the larger the membership of these samples will be, that means the larger the critical membership $\hat{\mu }$ should be, and vice versa. So, $\hat{\mu }$ can be defined as follows,

(7) $$\hat{\mu }=\frac{R}{d_{o}}$$

By the analysis above, the modified fuzzy membership function is defined as

(8) $$\mu_{i}=\{\begin{array}{ll} (1-\frac{R}{d_{o}})\times {\left(1-\frac{d(x_{i})}{R}\right)}^{1-\frac{d_{I}}{R}}+\frac{R}{d_{o}}, & d(x_{i})\leq R\\ \frac{R}{d_{o}}\times {\left(\frac{1}{1+(d(x_{i})-R)}\right)}^{K\times \frac{d_{o}}{R}}, & d(x_{i})>R \end{array}$$

In next section, an adaptive fuzzy membership based sparse representation classifier will be designed using the above modified fuzzy membership function for satellite cloud classification.

### 4.2. Classification of Clouds in Satellite Imagery Using Adaptive Fuzzy Sparse Representation

Sparsity is a common attribution of signals. Sparse representation means, in an appropriate base (dictionary), that a natural signal can be represented as a sparse linear combination of dictionary atoms; it is a concise way to represent information. In some sparse representation-based classification (SRC) algorithms [16,17,18], the sparse representation coefficients of a test sample can be obtained by sparse coding in a dictionary, and the test sample can be classified according to the sparsity and sparse concentration of representation coefficients. This paper applies SRC to the satellite cloud classification, to depict the fuzzy and uncertainty of cloud samples, and to eliminate the impact of outliers and noises for cloud classification. According to Equation (8), an adaptive membership of each training sample is calculated; based on the original feature vectors of each training sample, an adaptive fuzzy dictionary with an adaptive feature vector is constructed, which enhances the performance of sparse representation based classifiers for satellite cloud classification. 

As mentioned before, a 14-dimensional feature vector is extracted for each pixel, that is, for each sample in satellite cloud image. Denote $X=[X_{1},X_{2},\ldots ,X_{M}]$ as the training sample set with *M* cloud types, $X_{i}=[x_{1}^{\left(i\right)},x_{2}^{\left(i\right)},\cdots ,x_{j}^{\left(i\right)},\cdots ,x_{n}^{\left(i\right)}]\in {ℜ}^{m\times n}$ as the subset of the *i*-th type, $x_{j}^{\left(i\right)}\in {ℜ}^{m}$ as the feature vector of a training sample, where *m* = 14, i=1,2,…,M, j=1,2,…,n, *M* = 6 is the number of cloud types, and *n* = 100 is the number of training samples of the *i*-th type, the total number of training samples is l=M×n. For each training sample $x_{j}^{\left(i\right)}$, an adaptive membership $\mu_{i,j}$ can be calculated with Equation (8), and an diagonal matrix $U_{i}\in {ℜ}^{n\times n}$ can be constructed with $\mu_{i,j}$, j=1,2,…,n, as diagonal elements. The original training sample subset $X_{i}=[x_{1}^{\left(i\right)},x_{2}^{\left(i\right)},\cdots ,x_{j}^{\left(i\right)},\cdots ,x_{n}^{\left(i\right)}]$ of the *i*-th type can be weighted by $U_{i}$ to construct an optimized cloud training sample subset $D_{i}$,

(9) $$\begin{array}{ll} D_{i} & =X_{i}U_{i}\\ & =[x_{1}^{\left(i\right)},x_{2}^{\left(i\right)},\cdots ,x_{n}^{\left(i\right)}]\left[\begin{array}{cccc} \mu_{i,1} & & & \\ & \mu_{i,2} & & \\ & & \ddots & \\ & & & \mu_{i,n} \end{array}\right]\\ & =[d_{1}^{\left(i\right)},d_{2}^{\left(i\right)}\cdots ,d_{n}^{\left(i\right)}]=[\mu_{i,1}x_{1}^{\left(i\right)},\mu_{i,2}x_{2}^{\left(i\right)},\ldots ,\mu_{i,n}x_{n}^{\left(i\right)}]\in {ℜ}^{m\times n} \end{array}$$

here, $d_{j}^{\left(i\right)}=\mu_{i,j}x_{j}^{\left(i\right)},j=1,2,\ldots ,n$, $D_{i}=[d_{1}^{\left(i\right)},d_{2}^{\left(i\right)}\cdots ,d_{n}^{\left(i\right)}]\in {ℜ}^{m\times n}$. The same operation is performed on samples of all the *M* types, and the optimized training set is,

(10) $$D=[D_{1},D_{2},\ldots ,D_{M}]\in {ℜ}^{m\times l}$$

here, let D as the dictionary for sparse representation classifier. For a test sample $y\in {ℜ}^{m}$ from the *i*-th type, **y** could be expressed as a linear combination of those atoms $d_{j}^{\left(i\right)}$ from the *i*-th sub-dictionary $D_{i}$, that is $y=\alpha_{i,1}d_{1}^{\left(i\right)}+\alpha_{i,2}d_{2}^{\left(i\right)}+\cdots +\alpha_{i,n}d_{n}^{\left(i\right)}$, where $\alpha_{i,j},j=1,2,\ldots ,n$, are the coding coefficients. According to sparse representation theory, if **y** is represented as a linear combination of the entire dictionary D, only those coefficients corresponding to sub-dictionary $D_{i}$ will be nonzero. Thus, the above sparse representation can be modeled as:

(11) $$\begin{array}{ccc} \underset{\alpha }{\text{min}}\;{\left||y-D\alpha |\right|}_{2} & s.t. & {\left||\alpha |\right|}_{0}\leq E \end{array}$$

where E is a sparse threshold, $\alpha ={\left[\alpha_{1,1},\ldots \alpha_{1,n},\ldots ,\alpha_{i,1},\ldots \alpha_{i,n},\ldots ,\alpha_{M,1},\ldots \alpha_{M,n}\right]}^{T}$ is the sparse coefficient vector of y. The solution of Equation (11) is a NP-hard problem, and it is usually approximated by $\ell^{1}$-minimization,

(12) $$\begin{array}{ccc} \underset{\alpha }{\text{min}}\;{\left||\alpha |\right|}_{1} & s.t. & {\left||y-D\alpha |\right|}_{2}\leq \varepsilon \end{array}$$

where ε is an optional error tolerance. To calculate the sparse coding coefficient α, Equation (12) can be rewritten as the following general Lagrangian model:

(13) $$\alpha =\text{arg}\;\underset{\alpha }{\text{min}}\{{\left||y-D\alpha |\right|}_{2}^{2}+\lambda {\left||\alpha |\right|}_{1}\}$$

where λ is a positive constant, and a homotopy algorithm is used to solve the $\ell^{1}$-minimization problem [21]. A new operator $\delta_{i}(\alpha )$ is introduced to extract the entries in α that associate with the *i*-th type such as $\delta_{i}(\alpha )={\left[\alpha_{i,1},\alpha_{i,2},\ldots ,\alpha_{i,n}\right]}^{T}$, then, the test sample **y** can be reconstructed by the sub-dictionary $D_{i}$ as,

(14) $${\bar{y}}_{i}=D_{i}\delta_{i}(\alpha )$$

the reconstructed residual between y and ${\bar{y}}_{i}$ is:

(15) $$r_{i}(y)={\left||y-{\bar{y}}_{i}|\right|}_{2}$$

which indicates how best to represent **y** by the *i*-th sub-dictionary $D_{i}$. The smaller the value of $r_{i}(y)$ is, the more likely y belongs to the *i*-th type. So, the test sample y can be classified by seeking the minimum reconstructed residual.

(16) $$\text{identity}\;(y)=\text{arg}\;\underset{i}{\text{min}}\;\{r_{i}(y)={\left||y-{\bar{y}}_{i}|\right|}_{2}\},\;i=1,2,\ldots ,M$$

The proposed method for cloud classification is summarized as Algorithm 1:
**Algorithm 1**: Classification of clouds in satellite imagery using adaptive fuzzy sparse representation**Input:** a matrix of training samples $X=[X_{1},X_{2},\ldots ,X_{M}]\in {ℜ}^{m\times l}$ for *M* types, a test sample $y\in {ℜ}^{m}$;Normalize the columns of X to unit $\ell_{2}$-norm;Calculate the adaptive membership $\mu_{i,j}$ of each training sample from $X_{i}=[x_{1}^{\left(i\right)},x_{2}^{\left(i\right)},\cdots ,x_{j}^{\left(i\right)},\cdots ,x_{n}^{\left(i\right)}]\in {ℜ}^{m\times n}$ by Equation (8), construct the diagonal matrix $U_{i}\in {ℜ}^{n\times n}$, i=1,2,…,M, j=1,2,…,n;Construct an optimized cloud training sample subset $D_{i}$ as $D_{i}=X_{i}U_{i}$; conduct this processing for all the *M* types, and then construct the optimized training set $D=[D_{1},D_{2},\ldots ,D_{M}]\in {ℜ}^{m\times l}$;For test sample $y\in {ℜ}^{m}$, make D as the adaptive dictionary for sparse representation classifier, do $\ell^{1}$-minimization by solving Equation (17),
(17)$$\begin{array}{cccc} \hat{\alpha }=\text{arg} & \underset{\alpha }{\text{min}}\;||\alpha || & s.t. & {\left||y-D|\right|}_{2}\leq \varepsilon \end{array}$$
Compute the residuals:
(18)$$r_{i}(y)={\left||y-D_{i}\delta_{i}(\alpha )|\right|}_{2},\;i=1,2,\ldots ,M$$
**Output**: $\text{identity}\;(y)=\text{arg}\;\underset{i}{\text{min}}\;r_{i}(y)$, i=1,2,…,M

## 5. Simulation Results and Analysis

In this section, the performance of the proposed cloud classification system with some existing methods is compared. Experiments are conducted on a computer with 2.4 GHz Intel CPU and 4 GB RAM. The cloud image data come from the FY-2G satellite that carries five channel sensors (IR1, IR2, IR3, IR4, VIS) for a period of 10 days (from 28 June to 7 July 2016). From 28 June to 6 July 2016, the satellite data were collected at 8 a.m., 10 a.m., 12 p.m., 2 p.m., 4 p.m., and 6 p.m. Beijing time each day, so 9 × 6 = 54 times satellite data were collected. From these cloud images, three meteorologists examine the cloud images carefully, then manually identify and select 300 pixels (samples) for each of the six predefined cloud types as mentioned in Section 2.2. Totally, 1800 samples are used in experiments in Section 5.1 and Section 5.2. Satellite data collected at 2 p.m. Beijing time, on 7 July 2016, is used for experiment of visual comparison in Section 5.3. All experiments in Section 5.1, Section 5.2 and Section 5.3 use the same training samples. In order to effectively represent spectral information, feature vectors for each selected sample are extracted and normalized by $\ell_{2}$-norm. In the proposed AFSRC, the centers and radius *R* of hyperspheres for the membership value calculations were determined by SVDD, and Gaussian kernel function was used in SVDD. Constant C, as a penalty factor, which controls the trade-off between the volume and the errors in SVDD, is set to be C = 1.0. The classification accuracy of the proposed method is evaluated; several existing methods—including affinity based FSVM [14], SRC [16], and CCSI-ODSR [19]—are compared with the proposed method, the computation efficiency of the different methods is provided, and training time for training SVDD is listed in Section 5.4.

### 5.1. Accuracy Evaluation of AFSRC for FY-2G

Among the 300 pixels (samples) for each cloud type, randomly selected 100 pixels are used as training samples; the rest 200 pixels are used as testing samples. When parameter *K* = 5 in Equation (6), the confusion matrix of the classification results is given in Table 3. It can be seen that, among the 1200 test samples of 6 different cloud types, 1186 samples are correctly classified by the proposed AFSRC, the overall classification accuracy is 98.83%. For clear water and clear land, the classification accuracies achieved were 99.00% and 98.00%, respectively, which indicates the proposed AFSRC can identify these cloud-free areas effectively, and it can be applied in cloud detection in satellite cloud image analysis. For heap cloud and low cloud, the classification accuracy is 99.00% and 99.50%, respectively. Since heap clouds are often associated with strong convection weather and almost always leads to weather like lightning, showers, gustiness, or hailstones, while low clouds such as cumulus and nimbostratus are usually associated with continuous rainfall, these two clouds are the key monitoring objects of weather service, and their high recognition rates effectively show the application value of AFSRC. 

Table 4 shows the classification accuracy (%) of AFSRC with different *K*. It can be seen that the overall accuracy of AFSRC reaches its maximum with *K* = 5. When *K* varies, the classification accuracy of clear water and clear land remain almost unchanged, and the accuracy of other cloud types changes with a large range. Generally speaking, the overall accuracy of AFSRC with a smaller *K* is relatively lower, and it is relatively a bit higher with a larger *K*, which indicates that, with a larger *K*, the ability for sparse representation of the adaptive dictionary is strengthened, and the effect of outliers or noises can be suppressed. The next subsection will compare AFSRC with three competing methods.

### 5.2. Comparisons with Existing Methods

Some other methods have been applied in satellite cloud image classification recently; each method has its own advantages. In this experiment, AFSRC is compared with FSVM [14], SRC [16], and CCSI-ODSR [19]. As in the prior experiment, 200 samples of each cloud type are used for testing, and there are 1200 testing samples in total. The classification accuracy of the six cloud types by FSVM, SRC, CCSI-ODSR, and AFSRC are listed in Table 5.

It is clear that AFSRC achieves better results than the other methods for almost all cloud types, except AFSRC is slightly worse than affinity based FSVM for clear land. By introducing fuzzy membership, FSVM [14] can discriminate noises and outliers in training samples; its classification results are acceptable, except for low and medium cloud. As for SRC, testing samples of low cloud, medium cloud, and high cloud are often confused with each other, which leads to a poor result, the overall accuracy is less than 70%. The experiment result implies that SRC is not a reliable satellite cloud classification method. As for CCSI-ODSR, with the help of an over-complete dictionary, though the classification results for low cloud and high cloud are still improvable, the overall accuracy of CCSI-ODSR is much better. For AFSRC, by introducing an adaptive fuzzy membership function to eliminate the influence of noises and outliers, an optimized adaptive dictionary for sparse representation classifier is constructed, and the classification result is the best.

### 5.3. Benchmarks on FY-2G Satellite Data

**Figure 6:**
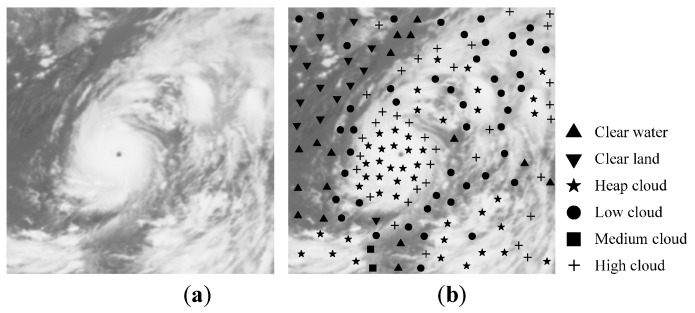
Original IR1 image and cloud types labeled image. (**a**) IR1 image; (**b**) Same image as (a) with cloud types labeled by a meteorologist.

In this experiment, the proposed AFSRC is benchmarked on the FY-2G satellite data obtained at 2 p.m. Beijing time, on 7 July 2016. From the satellite image of each channel, a specific sub-image with spatial resolution of 512 × 512 pixels, which covers the super typhoon “Nepartak” and parts of the southeast coast of china, is selected. The sub-image is used for a benchmark only; no testing sample is selected from this sub-image. The IR1 channel image and cloud types labeled image whose cloud types are identified by meteorologist are shown in Figure 6a,b, respectively. In Figure 6b, triangle (▲) indicates clear water, inverted triangle (▼) indicates clear land, star (★) indicates heap cloud, circle (●) indicates low cloud, square (■) indicates medium cloud, and cross (**+**) indicates high cloud.

**Figure 7:**
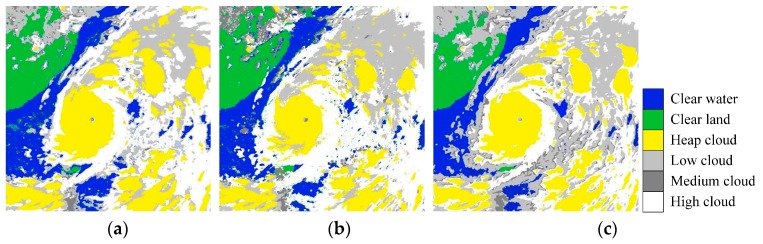
Cloud classification results by FSVM, CCSI-ODSR, and AFSRC. (**a**) FSVM; (**b**) CCSI-ODSR; (**c**) AFSRC.

Color-coded cloud classification images by FSVM [14], CCSI-ODSR [16], and AFSRC [19], are shown in Figure 7, whereas SRC is not included in this comparison due to its poor performance.

It can be seen from Figure 7 that, in most of the classification areas, the classification results of FSVM, CCSI-ODSR, and AFSRC are the same as that of the meteorologist-marking image. For example, for clear water and land, the three methods all give relatively reasonable classification results; in terms of the spiral rain-band cloud of super typhoon “Nepartak”, the three methods again mark it correctly. The upper left corner of Figure 6 is the Yangtze Plain area of China, where continuous heavy rain was causing a serious flooding disaster in early to mid of July 2016. In Figure 7, AFSRC and FSVM correctly identify the low clouds gathering in this area, which is consistent with the serious flooding disaster caused by the continuous heavy rain. On the left periphery of super typhoon “Nepartak”, FSVM misclassified a small portion of marine areas as land, and some low clouds and medium clouds around the typhoon body were misclassified as high clouds. In Figure 7b, compared with the classification results of FSVM and the proposed AFSRC, the area of heap clouds in typhoon body by CCSI-ODSR is much smaller than that of the other two. According to the discussions above, AFSRC can achieve better results for cloud classification; higher recognition accuracy for various cloud types indicates the strong stability and adaptability of AFSRC.

### 5.4. Running Time

To evaluate the computation efficiency of different methods, the training time and testing time of different methods are recorded, as shown in Table 6. For SRC, no training is needed; its training time is ignored. For each method, the training time is the total training time for all the 6 × 100 training samples, and the testing time is an average for all the 6 × 200 testing samples.

Compared with FSVM [14] and CCSI-ODSR [19], the training time of the proposed AFSRC is the shortest. The reason lies in that FSVM needs to calculate the fuzzy membership based on affinity among samples, which leads to a longer training time; CCSI-ODSR consumes a lot of time to train the dictionary, its high computational complex results in the longest training time; by introducing an adaptive fuzzy membership function to optimize the training samples and eliminate the impact of noises and outliers, the training time of AFSRC is shortened, too. Though the testing time of FSVM is much shorter than the other three, due to its relatively poor performance in misclassifying low cloud and medium cloud, and its poor adaptability and flexibility of the fuzzy membership function, it is hard for traditional FSVM to be applied in practice. Generally speaking, by constructing an adaptive fuzzy membership function, the proposed AFSRC achieves high classification accuracy with acceptable time efficiency, and is reliable in practical cloud classification.

## 6. Conclusions

Since traditional fuzzy membership has poor flexibility and is inconsistent with the distribution characteristics of samples for cloud classification, by defining adaptive parameters related to attenuation rate and critical membership, an adaptive fuzzy membership function is constructed and then combined with sparse representation classifier. The newly proposed adaptive fuzzy sparse representation-based classification (AFSRC) method for satellite cloud classification can eliminate the impact of noises and outliers for cloud classification. Experiments results on FY-2G satellite cloud images show that the proposed AFSRC not only improves the accuracy of cloud classification with acceptable time efficiency, but it also has strong stability and adaptability. How to find a more reasonable way to determine the parameters in AFSRC, and how to apply the AFSRC method in other satellite cloud classification, will be presented in future work.

## Figures and Tables

**Table 1:** The components of feature vector for cloud classification.

Component	Description
G1, G2, G3, G4, GV	Gray value of IR1, IR2, IR3, IR4, VIS
T1, T2, T3, T4	Brightness temperature of IR1, IR2, IR3, IR4
A	Albedo of VIS
T1-T2, T1-T3, T1-T4, T2-T3	Brightness temperature difference IR1-IR2, IR1-IR3, IR1-IR4, IR2-IR3

**Table 2:** The main identification characteristics of different components.

Component	Identification Characteristics
G1, G2, T1, T2	Can be used to identify land, ocean, and clouds
G3, T3	Can be used to measure the water vapor content of clouds
G4, T4	Mainly represent the characteristics of under clouds over the ocean
GV, A	Mainly represent the thickness, height, and composition of clouds
T1-T2	Mainly describe the characteristics of cirrus and cumulonimbus
T1-T3, T1-T4, T2-T3	Indicate the height of clouds more precisely

**Table 3:** Confusion matrix of classification results of different cloud types by AFSRC (K = 5).

Cloud Type	Classified as					
	Clear Water	Clear Land	Heap Cloud	Low Cloud	Medium Cloud	High Cloud
Clear water	198	2	0	0	0	0
Clear land	3	196	0	1	0	0
Heap cloud	0	0	198	1	0	1
Low cloud	0	0	0	199	1	0
Medium cloud	0	0	0	1	198	1
High cloud	0	0	0	3	0	197

**Table 4:** Classification accuracy (%) of different cloud types by AFSRC using different value of K.

K	Clear Water	Clear Land	Heap Cloud	Low Cloud	Medium Cloud	High Cloud	Overall Accuracy
K = 0.5	98.50	98.00	94.50	90.00	88.00	92.50	93.58
K = 1	98.00	97.00	96.00	89.00	92.50	96.00	94.75
K = 3	99.00	97.50	95.00	95.00	97.00	95.50	96.50
K = 5	99.00	98.00	99.00	99.50	99.00	98.50	98.83
K = 7	98.00	98.50	96.50	99.00	93.00	95.00	96.67
K = 9	97.00	97.00	97.50	98.50	93.00	95.50	96.42
K = 11	97.50	97.00	96.50	98.00	94.00	96.50	96.58

**Table 5:** Classification accuracy (%) of different cloud types by four methods.

Method	Clear Water	Clear Land	Heap Cloud	Low Cloud	Medium Cloud	High Cloud	Overall Accuracy
FSVM	97.50	99.50	98.50	90.50	91.50	97.50	95.83
SRC	83.50	70.50	91.00	62.50	52.00	58.50	69.67
CCSI-ODSR	98.50	96.00	91.50	88.00	97.00	91.50	93.75
AFSRC	99.00	98.00	99.00	99.50	99.00	98.50	98.83

**Table 6:** Training/testing time of different methods.

Method	FSVM	SRC	CCSI-ODSR	AFSRC
Training time (s)	1.92	Null	8.61	1.01
Testing time (ms)	0.13	4.47	10.06	3.82
